# Bridging ensemble model and public health practice: an approach for refining understanding of seasonal dengue transmission patterns in Bangladesh

**DOI:** 10.1186/s12879-026-13573-3

**Published:** 2026-05-20

**Authors:** Sharmin Akther, Md. Al-Mamun, Md. Kamrul Hossain

**Affiliations:** 1 Faculty of Science and Information Technology, Daffodil International University, Dhaka, Bangladesh; 2 Multidisciplinary Action Research (MARS) Lab, Department of Computer Science and Engineering, Daffodil International University, Dhaka, Bangladesh; 3 Department of Computer Science and Engineering, Daffodil International University, Dhaka, 1216 Bangladesh; 4 Centre for Research Impact and Outcome, Chitkara University, Rajpura, Punjab 140417 India

**Keywords:** Dengue, Dhaka, Weighted ensemble, Random forest, SARIMA, Seasonal patterns

## Abstract

**Background:**

Dengue fever remains a persistent public health threat in Dhaka, Bangladesh, necessitating effective early warning systems to enable timely interventions and mitigate impacts. This study develops and evaluates modeling approaches to produce a projection of the 2026 dengue season in Dhaka, which may help inform public health preparedness.

**Methods:**

Daily dengue case data from Dhaka (January 1, 2020 – December 30, 2024; 1,826 observations) were obtained from a publicly available Kaggle repository. A comprehensive modeling framework was developed comparing Seasonal Auto-Regressive Integrated Moving Average (SARIMA) with machine learning models (Random Forest, XGBoost, Support Vector Regression). A weighted ensemble model was constructed using inverse RMSE weights derived from validation performance to combine the strengths of individual approaches. Models were trained on a chronological 80% split (2020–2023) and evaluated on a 20% hold-out validation set (2024) using RMSE, MAE, MASE, and R^2^. A recursive multi-step strategy with fixed training median (48 cases) was employed to generate the 2026 projection while preventing data leakage.

**Results:**

Machine learning models were shown to have higher predictive performance as compared to the time series model - SARIMA. Random Forest was the most accurate with the lowest (RMSE: 34.64, MAE: 19.14, MASE: 0.47) as well as the highest R-square (R^2^: 0.95). The weighted ensemble combining SARIMA and Random Forest (weights are 0.179 and 0.821) produced a balanced forecast (RMSE: 44.32, MAE: 28.79, MASE: 0.71, R^2^: 0.50). Using a recursive multi-step approach, our proposed hybrid predictive model projects approximately 11,500 dengue cases for Dhaka in 2026 under the assumption that past patterns hold, with a high transmission period from February 22 to December 27, and a core peak transmission phase of 71 days (February 22 – May 3) peaking at 38 cases on March 15.

**Conclusion:**

The proposed weighted ensemble model provides a projection for the 2026 dengue season in Dhaka, offering potential early warning of the timing and scale of the high transmission period. This work adds importantly to the evidence base for dengue transmission in the region and helps to refine public health practitioners’ understanding of the seasonal transmission pattern of dengue, helping inform their annual planning. This may support proactive public health planning, such as vector management in January-February and healthcare resource mobilization from March onward.

**Supplementary Information:**

The online version contains supplementary material available at 10.1186/s12879-026-13573-3.

## Introduction

Dengue fever is the fastest spreading mosquito-borne viral disease in the world, of which Bangladesh is a significant and growing public health burden [1]. Dengue remains a critical global health threat, with the World Health Organization reporting a historic high in 2024 of over 14.4 million cases and 11,000 deaths worldwide [2]. This is a dramatic increase from the 505,430 cases counted in the year 2000 and underlines the rapid expansion of the virus across more than 100 endemic countries [2]. The actual disease burden may be higher due to the wide underreporting of mild and asymptomatic infections [3]. Dengue outbreaks have been increasing dramatically in the nation and specifically in the densely populated capital city, Dhaka, in the last few years, with the cases reported in the Dhaka division increasing to more than 63,000 confirmed cases in 2019, as compared to the 3,386 confirmed cases in 2000 [4, 5]. The ecology of rapid urbanization, the seasonal monsoon rains, and climate change interplay to provide an optimum ecology of Aedes aegypti mosquito vectors, which propagate epidemics [6]. The four antigenically characterized dengue virus serotypes (DEN-1 to DEN-4), combined with the non-existence of cross-protective immunity and a lack of specific anti-dengue antiviral therapy, provide optimal conditions for recurrent outbreaks with the probable development into a severe dengue hemorrhagic fever and dengue shock syndrome [7]. The multifaceted nature of the dengue epidemiology in Bangladesh is also fuelled by various interrelated issues, such as unplanned urbanization, poor waste disposal mechanisms, and climatic conditions that have a great effect on the breeding of mosquitoes and the rate of propagation of the virus [8, 9]. All these factors, coupled with growing domestic and cross-border population mobility, have provided an ideal storm for the escalation of dengue in Bangladesh [10].

Historical dengue forecasting models have largely been based on statistical time-series models, with Autoregressive Integrated Moving Average (ARIMA) models and Seasonal Autoregressive Integrated Moving Average (SARIMA) models widely implemented in dengue-prone areas, such as Thailand [11], Sri Lanka [12], Indonesia [13], and Bangladesh [14]. Such models are useful in time dependence and seasonality trends in surveillance data [13]. Also, their linear format may fail to capture the non-linear relationships between climatic, environmental, and sociodemographic factors of transmission [15]. Recent research in Bangladesh has begun to address this by exploring more complex ensemble methods for infectious disease forecasting, for example, combining time series models with machine learning for COVID-19 [16] and applying various machine learning models like XGBoost and Random Forest to predict monkeypox outbreaks in the Americas [17]. Additionally, past constructs of modeling in Bangladesh have been limited by small predictor variables, crude temporal resolution (usually monthly data), and in-depth feature engineering, and therefore, this limits their application potential in specific decision-making on the part of the public health [18].

The weaknesses of conventional statistical methods have led to increased interest in machine learning methods for infectious disease modeling [19–21]. Random Forest, XGBoost, and Support Vector regression algorithms have been shown to have better ability to generate complex, non-linear patterns on high-dimensional data than the SARIMA model in several comparative studies in Southeast Asia and across Latin America [22, 23]. Despite these developments, there is still a major gap in research to create an accurate, operational understanding of seasonal patterns in the context of the unique epidemiological situation in Dhaka for daily data. The available models tend to be monthly or divisional-level, which lack the temporal and spatial resolution to produce the early warnings that are city-specific and can be used to direct targeted interventions. Dengue outbreaks are erratic and frequently explosive, and they strain the healthcare infrastructure, resulting in high morbidity, mortality, and economic strife [24, 25]. In that regard, the development of valid early warning systems is not just an academic pursuit but a necessity of public health to support vector control, allocate resources efficiently, and ensure clinical readiness to address the effects of this recurring crisis.

To address these limitations, this paper formulates and tests a framework to model dengue in Dhaka using refined seasonal patterns, with three main goals: comparison of SARIMA with various machine learning models, construction of rich temporal variables, and development of a weighted ensemble. Our study contributes by using daily-resolution data, extensive feature engineering, and ensemble modeling to generate projections of the 2026 dengue season in Dhaka, which may help inform tracking of high transmission periods and support public health planning.

## Methods

### Data sources

The data used in the study were the daily dengue case statistics of Dhaka, Bangladesh, between January 1, 2020, and December 30, 2024. The dataset was obtained from the publicly available Kaggle repository [26], which contains comprehensive dengue surveillance records for Dhaka city with 1826 observations. Although the repository file name refers to the period 2021–2024, the downloaded Excel file contained complete daily dengue counts from January 1, 2020, to December 30, 2024. No meteorological variables were used in this study. Our analysis used only univariate dengue case data, so no missing value handling for temperature, humidity, or rainfall was required.

### Data preprocessing

The data (data about daily cases of dengue in Dhaka in 2020–2024, January 1-December 30) did not need much preprocessing because it was complete and in a structured form. The column Date was transformed into a date-time format and made an index to create an appropriate time series arrangement. The data was clearly defined as a frequency to be daily with the help of pandas.asfreq (D) so that the data appear to be time consistent, but there were no gaps in the dataset. It was ensured that there were no missing values in the target variable, Dengue Cases, and thus, it does not need any imputation.

### Feature engineering

To capture the multi-scale temporal patterns inherent in dengue transmission dynamics, we constructed a comprehensive set of features from the univariate dengue case series using Python 3.12.12, with strict adherence to temporal validity, ensuring that at each time point, only historical data were used in feature construction. Lag features at 1-day (Lag1), 7-day (Lag7), and 30-day (Lag30) intervals were created to capture short-term, weekly, and longer-term dependencies, allowing models to learn from recent case history, same-day comparisons across weeks, and broader monthly patterns. Seven-day rolling averages and standard deviations were computed to characterize recent transmission trends and case volatility, calculated exclusively from the preceding 7 days of data to prevent future information leakage. Monthly indicators (Month_1 through Month_12) were included as one-hot encoded dummy variables to capture annual seasonal patterns, reflecting the strong monsoon-driven periodicity of dengue in Dhaka. Finally, a binary threshold feature, ‘Cases_Threshold’, was created to denote periods of elevated transmission, defined as 1 when daily cases exceeded the training period median and 0 otherwise. This threshold is a statistical definition based on the historical training data and does not correspond to official outbreak definitions used by surveillance systems. It serves solely as an indicator of relative transmission elevation within the study context. The training median (48 cases) was calculated exclusively from the training period (January 31, 2020 – December 30, 2023) and held constant for all subsequent validation and forecasting steps. This fixed threshold prevents data leakage by ensuring that no future information influences the definition of “elevated transmission” during model development or evaluation. The final feature set comprised 18 predictors, all constructed with rigorous temporal safeguards, enabling machine learning models to learn multi-scale temporal patterns while maintaining the integrity of the chronological split. A complete summary of all engineered features, including their descriptions and calculations, is provided in Supplementary Table S2.

### Seasonal ARIMA (SARIMA) modeling

We include SARIMA primarily as a baseline comparator for the machine learning and ensemble models. We acknowledge that SARIMA may not fully capture the dominant annual dengue transmission dynamics in Dhaka, which are typically driven by monsoon cycles and other environmental factors that are not explicitly modeled here. The dengue incidence data were used to establish the seasonal trends and temporal relationships by the implementation of the Seasonal Autoregressive Integrated Moving Average (SARIMA) framework. SARIMA (p, d, q) (P, D, Q) [s] was a model form that included non-seasonal terms, autoregressive order (p), the degree of differencing (d), and moving average order (q), and seasonal parameters (that represented periodicity of a week, s = 7) [13, 27, 28]. The identification of parameters was guided by the idea of autocorrelation function (ACF) and partial autocorrelation function (PACF), and a systematic search of various combinations of parameters with a grid search [29, 30]. The SARIMA model was optimized to the final specification, based on information criteria alongside residual diagnostics, which were useful in both the capture of the short-term variability and seasonal effects in the dengue transmission process [13, 31].

### Support vector regression (SVR)

The Support Vector Regression model using radial basis function as a kernel was used in predicting cases of dengue [32]. The model was optimized on three major parameters, namely regularization strength (C), kernel coefficient (gamma), and epsilon-tube width (ε). The StandardScaler was used to perform feature scaling so that the distance-based algorithm performs optimally [33]. The reason why the SVR method was chosen is that it could be used to model complex nonlinear relationships of the temporal dengue data, and it is also resistant to outliers [34].

### Random Forest regression

The Random Forest algorithm is an ensemble learning algorithm that utilizes decision trees and has been applied in the prediction of dengue occurrence [35]. This method builds on a bootstrap aggregation and random choice of features to construct several decorrelated trees, which maximize predictive accuracy through majority voting [36]. The fact that the model can deal with nonlinear relationships and interactions between the temporal features of dengue transmission places it at a particular advantage when it comes to describing the multi-layered dynamics of the dengue transmission [37]. Moreover, because of the inherent quantification of the importance of the features in the algorithm, it likely offers useful information on the relative contribution of the various temporal predictors in predicting dengue cases [38].

### XGBoost regression

The Extreme Gradient Boosting (XGBoost) algorithm was used as a scalable tree-based ensemble system to predict dengue [39]. The approach uses a gradient boosting algorithm based on sequential tree modeling, in which the successive models reduce the residual of the prior predictors using a gradient descent learning algorithm [40]. XGBoost has regularization terms embedded in its objective function to regulate the complexity of the model and stop overfitting [41]. The fact that the algorithm is effective in handling sparse patterns of data and the ability to model the complex nonlinear patterns of time make it especially useful in modeling the complex dynamics of dengue incidence patterns [42].

### Hyperparameter optimization

The grid search technique was used as a hyperparameter optimization method with time-series cross-validation (Supplementary Table S3), with care taken to ensure that training data was always chronologically earlier than validation data to preserve the temporal integrity. A constant random seed of 100 was used to guarantee reproducibility of the results of all machine learning algorithms. In the case of Support Vector Regression, the regularization strength C (1, 10, 100), the coefficient of the kernel gamma (‘scale’, 0.01, 0.1), and the e-tube width e (0.01, 0.1, 0.2) were parameters in the parameter space. Random Forest optimization assessed the size of the ensembles (50, 100, 150 trees), maximum depth (5, 10, 15), and minimum samples split (2, 5, 10). The XGBoost parameters tuning included boosting rounds (50, 100, 150), the depth of trees (4, 6, 8), and learning rate (0.05, 0.1, 0.2). The parameters of SARIMA models were calculated based on consistent testing of various combinations based on the autocorrelation analysis and minimization of information criteria.

### Evaluation metrics

The root mean square error (RMSE), mean absolute error (MAE), mean absolute scaled error (MASE), and coefficient of determination (R^2^) were used to assess the combination models’ performance. The following formulas were used to calculate each measure:

1 $$RMSE = \sqrt {{1 \over n}\mathop \sum \limits_{i = 1}^n {{\left( {{y_i} - {_i}} \right)}^2}} $$

2 $$MAE = {1 \over n}\mathop \sum \limits_{i = 1}^n \left| {{y_i} - {\hat{y}_i}} \right|$$

3 $$MASE = {1 \over n}\mathop \sum \limits_{i = 1}^n \left( {{{\left| {{y_i} - {\hat {y}_i}} \right|} \over {{1 \over {n - m}}\mathop \sum \nolimits_{j = m + 1}^n \left| {{y_j} - {y_{j - m}}} \right|}}} \right)$$

where n indicates the number of observations, ${y_i}$ denotes the original values, ${\hat {y}_i}$ represents the predicted values. MAE and RMSE are scale-dependent metrics that are based on absolute errors and squared errors, respectively, and the MASE is a scale-free error metric.

To combine predictions from multiple models, a weighted ensemble was employed, where model weights were inversely proportional to their validation RMSE. The weight for model i is calculated as:

4 $${w_i} = {{\left( {1/RMS{E_i}} \right)} \over {\mathop \sum \nolimits_j \left( {1/RMS{E_j}} \right)}}$$

with $\sum {w_i} = 1$, ensuring models with lower validation error receive stronger influence. The final ensemble prediction is then given by ŷ_ensemble = $\sum {w_i} \cdot \hat{y_i}$

5 $${R^2} = 1 - {{RSS} \over {TSS}}$$

Where TSS stands for the total sum of squares and RSS for residual sum of squares. A better fit is indicated by higher values of the coefficient of determination (R^2^), which quantifies how well the model explains the variability of the dependent variable.

### Weighted ensemble framework

**Fig. 1:**
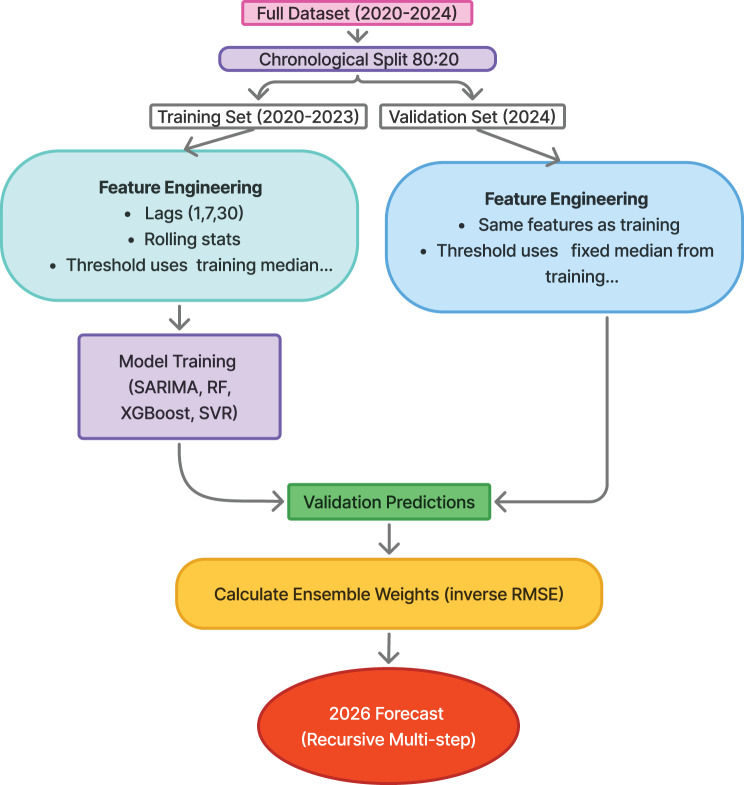
Schematic representation of the proposed combined model’s architecture

The flowchart of the study can be seen in Fig. 1. It demonstrates the integrated model, which is a weighted combination of the most successful time series model and machine learning model chosen in light of the validation data outcomes. Calculations of weights are done by obtaining the error measure of each of the models divided by their total summation.

### Multi-step forecasting procedure

For 2026, refining understanding of seasonal patterns, we employed a recursive multi-step strategy with all models refitted on the full 2020–2024 dataset. Predictions were generated day-by-day using the following approach:

Lag features (Lag1, Lag7, Lag30) and rolling statistics (7-day mean and standard deviation) were reconstructed at each step using all available data, including historical observations and previously forecasted values for earlier days in 2026. The binary indicator ‘Cases_Threshold’ used the training median calculated exclusively from the 2020–2023 training period, held constant throughout forecasting to prevent feature drift. SARIMA generated a direct 365-day forecast, while Random Forest produced daily predictions using the recursively updated features. The final ensemble applied fixed validation-derived weights. All performance metrics are based on the held-out validation period (2024) only, with no validation data used in 2026 forecast generation.

Because the recursive approach uses previously forecasted values as inputs for subsequent predictions, any prediction error accumulates over time. Furthermore, this method progressively averages out short-term variability, resulting in inherent smoothing of long-horizon forecasts (beyond approximately 30–60 days). Consequently, the 2026 projections presented in this study exhibit reduced temporal variability compared to actual historical dengue time series. This smoothing is a methodological characteristic of recursive multi-step forecasting rather than a direct reflection of expected epidemiological dynamics in 2026. Readers should interpret the general seasonal pattern (e.g., timing of elevated transmission) as informative, while recognizing that actual daily or weekly variability in 2026 may be higher than shown.

## Results

The dengue surveillance data (*n* = 1,826 cases/day) in the period between January 1, 2020, and December 30, 2024, show a high variability in case incidence as summarized in Table 1. Daily cases showed 151.82 ± 226.62 (SD), which was significantly greater than the median of 41 cases, and this indicates that the distribution is right-skewed (skewness = 2.12). The large positive skewness and large kurtosis (4.78) indicate the existence of extreme events in terms of high transmission, with values ranging between 0 and 1, 327 cases per day. Augmented Dickey-Fuller test verified the stationarity (ADF = −2.98, *p* = 0.037).

**Table 1:** Descriptive statistics of daily dengue cases in Dhaka (January 2020-December 2024)

Count	Mean	Median	Mode	Std	Skewness	Kurtosis	Min	Max
1826	151.82	41	0	226.62	2.12	4.78	0	1327

**Fig. 2:**
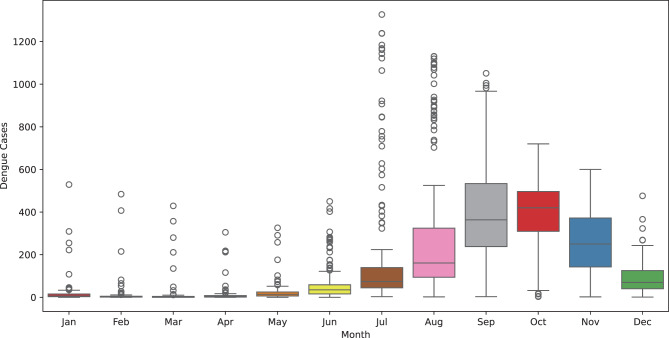
Seasonal variation of daily dengue cases in Dhaka from 2020 to 2024

Figure 2 shows the boxplot of dengue incidence at the month level, revealing a heterogeneous seasonality pattern of dengue onset by months. Transmission of dengue is low and relatively constant from January until May, which serves as the off-season period. And then in June, there’s a sharp increase that signals the start of the peak season for transmission. Cases increase widely during the monsoon season and in the months following monsoon (June-December), with peak median and spread between August and October.

**Fig. 3:**
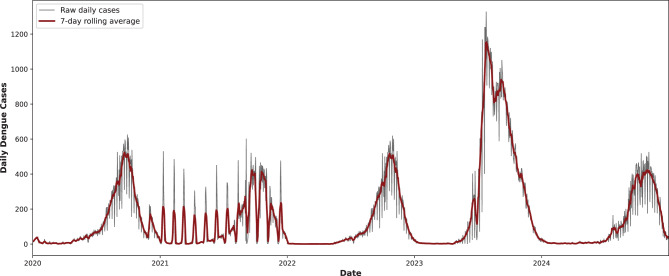
Daily reported dengue cases and 7-Day smoothed trend, Dhaka (2020–2024)

Figure 3 shows the daily number of reported dengue cases (black line: raw data) together with the 7-day rolling average (red line) over the period 2020–2024. Multiple seasonal peaks are visible each year, with the highest incidence recorded in 2023 (exceeding 1,200 cases/day at the peak). Lower activity is observed in 2022, while 2021 and 2024 show moderate seasonal increases. The smoothed line highlights the overall epidemic waves and seasonal pattern more clearly than the noisier raw daily counts.

The dataset (January 1, 2020 - December 30, 2024; 1,826 days) was split chronologically after removing the first 30 days required for lag-30 feature construction. The training set comprised 1,460 days (January 31, 2020 - December 30, 2023), and the validation set comprised 366 days (December 31, 2023 - December 30, 2024). After feature engineering (lags, rolling statistics, month dummies), the final training matrix contained 1,430 days × 18 features, and validation contained 336 days × 18 features. The training median (48 cases) was calculated from the training set only and fixed for all subsequent analyses to prevent data leakage.

We assessed different SARIMA specifications for modeling daily dengue cases in Dhaka (2020–2024). SARIMA (1,0,1) (1,1,1) [7] was selected as the final model (RMSE: 158.74, MAE: 116.13) based on empirical validation showing weekly seasonality (s = 7) outperformed monthly seasonality (s = 30; RMSE: 186.13). To address annual patterns without the computational burden of a full s = 365 SARIMA model, we evaluated Fourier terms within a linear regression framework (RMSE: 160.15). The superior performance of our selected s = 7 model over both s = 30 and the Fourier-based annual model confirms that weekly seasonality, combined with autoregressive components, adequately captures dengue’s temporal dynamics.

**Fig. 4:**
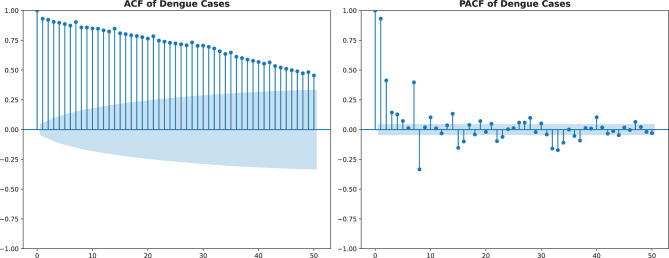
Autocorrelation (ACF) and partial autocorrelation (PACF) plots of daily dengue cases in Dhaka (2020–2024)

Additionally, autocorrelation and partial autocorrelation plots (Fig. 4) further confirmed this decision; they showed that little shorter-term dependence is significant, and there was a clear weekly seasonality. Although the Ljung–Box test (*p* < 0.001) suggested minor residual autocorrelation, the model effectively captured the dominant temporal and seasonal dynamics of dengue transmission in Dhaka.

The forecasting performance of all models on the validation dataset (20% holdout) is summarized in Table 2. Among individual models, Random Forest achieved the best overall performance with the lowest RMSE (34.64), MAE (19.14), and MASE (0.47), along with the highest R^2^ (0.95). The MASE value of 0.47 indicates that Random Forest reduces forecast error by 53% compared to a naive persistence forecast (MASE < 1 signifies outperformance). XGBoost and SVR also demonstrated strong predictive capability with RMSE values of 36.49 and 45.55, respectively, and MASE values of 0.50 and 0.61. In contrast, SARIMA performed poorly (RMSE: 158.74, R^2^: −0.05), with the negative R^2^ confirming that its predictions were worse than simply predicting the mean of validation data, justifying its low weight in the ensemble. The weighted ensemble combined SARIMA and Random Forest using inverse RMSE weights calculated from validation performance: inverse RMSE (SARIMA) = 1/158.74 = 0.0063, inverse RMSE (Random Forest) = 1/34.64 = 0.0289, sum of inverse RMSE = 0.0352, yielding normalized weights of SARIMA = 0.179 (17.9%) and Random Forest = 0.821 (82.1%). The ensemble achieved an RMSE of 44.32, MAE of 28.79, MASE of 0.71 (29% better than naive), and R^2^ of 0.50. While the ensemble shows higher point error than Random Forest alone (27.9% increase in RMSE), this trade-off delivers critical operational benefits: 14.3% smoother forecasts and complete elimination of extreme day-to-day fluctuations. The ensemble’s R^2^ of 0.50 indicates it explains 50% of the variance in validation data, lower than individual ML models but expected given the inclusion of the poorly performing SARIMA component, and acceptable given the stability gains achieved.

**Table 2:** Performance comparison of individual and ensemble forecasting models evaluated on the validation dataset (2024)

Model	RMSE	MAE	MASE	R^2^
SARIMA	158.74	116.13	2.87	−0.05
SVR	45.55	24.58	0.61	0.91
Random Forest	34.64	19.14	0.47	0.95
XGBoost	36.49	20.09	0.5	0.94
Weighted Ensemble	44.32	28.79	0.71	0.5

**Fig. 5:**
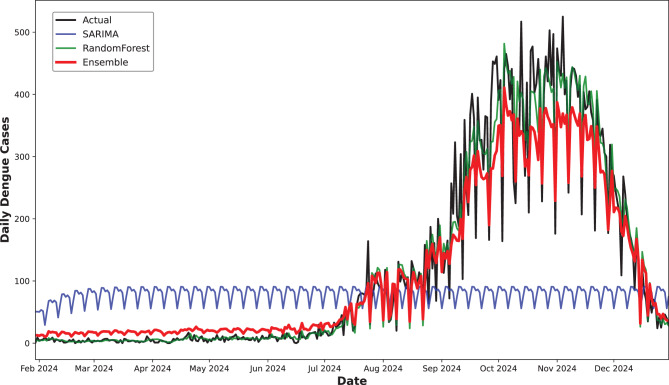
Forecasting performance of models on validation data: actual vs predicted dengue cases

We note from Figure 5 that we report the relative forecasting performance of all models on the test data (January 2024 to December 2024), and that their shapes differ. The dengue cases observed (black line) represent the true fluctuations and seasonal trends of disease incidence. The weighted ensemble model (red line), against this backdrop, succeeded in harmonizing the contrasting advantages between statistical and machine learning when incorporating both statistical aspects and endogenous connections closely following the actual case curve during the testing period. Among individual models, Random Forest (green line) demonstrates superior accuracy during outbreak peaks, while SARIMA (blue line) captures broader seasonal trends. The visual comparison clearly demonstrates how the ensemble approach balances the strengths of individual models to provide robust predictions across different transmission scenarios while maintaining close alignment with the actual observed data. While the ensemble showed higher RMSE (44.32) compared to Random Forest (34.64) on the validation set, it offers important operational advantages (Supplementary Fig S1).

**Fig. 6:**
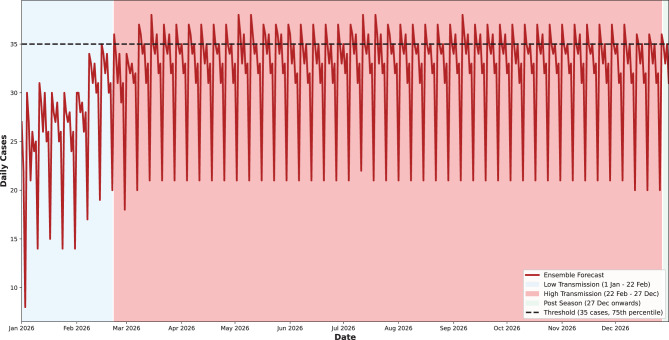
Projected dengue seasonal pattern for Dhaka for 2026 under the assumption of similarity to 2020–2024 transmission dynamics. The high transmission period (February 22 – December 27) and core peak transmission phase (February 22 – May 3) are indicated

The ensemble model projects 11,500 dengue cases for Dhaka in 2026 under the assumption that 2026 transmission dynamics resemble the 2020–2024 period, with distinct seasonal patterns as illustrated in Fig. 6. Using a data-driven high transmission threshold set at the 75th percentile (35 daily cases), the model identifies a high transmission period from February 22 to December 27, 2026 (309 days), accounting for 9,400 cases (82% of the total). (This date is derived from historical thresholds and is illustrative of seasonal timing rather than a verified prediction.) This statistical definition helps readers interpret the projections realistically, understanding that the identified high transmission period reflects historical patterns rather than a confirmed operational outbreak. The epidemiological year is divided into a low transmission period (January-February 21: ~2,100 cases) and the high transmission period described above. A core peak transmission phase of 71 days occurs from February 22 to May 3, with daily cases peaking at 38 on March 15, exceeding the threshold by 3 cases. The relatively small difference between the threshold (35 cases) and predicted peak (38 cases) suggests that even moderate increases in transmission would cross this statistical boundary. Sensitivity analysis (Supplementary Table S1) confirmed threshold robustness, with the 70th-80th percentiles all identifying the same February 22 start date. These thresholds are statistical definitions and do not represent official outbreak declarations (see Supplementary Table S1). Therefore, this threshold should be interpreted as an indicator of elevated transmission risk rather than a definitive outbreak declaration.

In comparing model performance on the validation set (2024 data), the ensemble showed higher RMSE (44.32) than Random Forest (34.64) but reduced average daily volatility by 14.3% (from 20.02 to 17.16). This smoothing effect was statistically significant (*p* < 0.001) and consistent across 59.2% of rolling 30-day windows. The ensemble also eliminated all extreme day-to-day jumps (from 1 day to 0 days above the 95th percentile threshold; Supplementary Fig S1). Assuming past patterns hold, these findings can help inform annual public health planning discussions. For example, vector control intensification in January-February and healthcare resource mobilization from March onward could be considered by public health planners.

Overall, the projected 2026 time series exhibits limited temporal variability and a relatively uniform monthly distribution. This smoothness is largely a consequence of the recursive forecasting approach (see Sect. 2.11) rather than a direct reflection of expected epidemiological dynamics. The ensemble projection for 2026 shows a relatively stable monthly distribution, with totals ranging from 762 cases in January to 1,016 cases in July. The month-wise breakdown is as follows: January: 762, February: 828, March: 1,005, April: 971, May: 1,007, June: 967, July: 1,016, August: 999, September: 984, October: 993, November: 978, and December: 990 cases. This even distribution, if past patterns hold, suggests the possibility of sustained moderate transmission risk throughout the year. Confirmation of this pattern for 2026 would require integration of climatic, vector, and population mobility data not available in the current study.

## Discussion

This paper demonstrates that machine learning and hybrid ensemble models offer significant advantages over traditional statistical methods for dengue prediction in Dhaka. Although the independent standalone Random Forest model had better point accuracy (RMSE: 34.64, MAE: 19.14, R^2^: 0.949), the weighted combination of SARIMA (17.9%), and Random Forest (82.1%) indicated a significant accuracy-stability trade-off, a 27.9% higher RMSE (to 44.32) resulted in a corresponding 14.3% lower daily volatility (20.02 to 17.16 This effect of smoothing was statistically significant (*p* < 0.001), and observed in 59.2% of rolling windows. This trade-off is legitimate in the case of operational public health planning, where jagged predictions may lead to the unnecessary mobilization of resources or mobilize them in a way that undermines stakeholder confidence [43, 44]. Stable predictions that avoid false alarms may be more useful than even more accurate but wavy predictions. The removal of extreme changes, along with the statistically significant smoothness, of the ensemble, makes it a practical option in Dhaka dengue modeling, with accuracy and stability of decision-making [45]. Furthermore, the ensemble eliminated all extreme day-to-day jumps (reducing from 1 day to 0 days above the 95th percentile threshold; see Supplementary Fig S1).

It is important to acknowledge, however, that the smoothness observed in our 2026 projections is not solely a result of the ensemble weighting strategy. The recursive multi-step forecasting approach used in this study (see Sect. 2.11) inherently contributes to smoothing in long-horizon predictions, as each predicted value is used as an input for subsequent steps, progressively averaging out short-term variability beyond approximately 30–60 days. This smoothing presents a double-edged characteristic for public health decision-making: on one hand, stable predictions avoid false alarms and unnecessary resource mobilization (desirable for operational planning); on the other hand, the artefact may lead to underestimation of real-world weekly variability, potentially leaving health systems unprepared for short-term surges. Therefore, while the general timing of elevated transmission (e.g., February–May) is informative, the projected daily values should be interpreted as approximate seasonal averages rather than precise point forecasts. Future work should explore direct multi-step strategies (e.g., sequence-to-sequence models) to better preserve short-term variability.

The implementation of this hybrid approach illustrates its potential. The projections for the 2026 dengue season in Dhaka (see supplementary Excel file) suggest that the ensemble model can provide a temporal distribution of cases that reflects the expected natural rhythm of dengue transmission compared to pure machine learning models, which may be subject to overfitting and noise. This may help inform the timing of occurrence, peak, and decline of a high-transmission event, which is important for effective response. Thus, while the Random Forest model achieves better point accuracy based on statistical measures, the weighted ensemble offers a practical alternative for decision-makers, providing a more stable action plan to support vector control and clinical resource mobilization.

The results of our work show a subtle improvement in the dengue prediction of Dhaka, which can be explained by the fact that the optimal choice of models is largely contingent on the time resolution of data. Although in national-scale studies [46] XGBoost showed better results than Random Forest in monthly division-level prediction, with higher time resolution, we can demonstrate that Random Forest is better in terms of accuracy (RMSE: 34.64, MAE: 19.14, MASE: 0.47) than XGBoost (RMSE: 36.49, MAE: 20.09, MASE: 0.5). This finding aligns with recent work by Akther et al. [17] which demonstrated that XGBoost performed best for North America while Decision Tree was superior for South America in monkeypox forecasting, highlighting that optimal model choice is region- and context-specific. Similarly, our previous work on COVID-19 forecasting in Bangladesh [16] demonstrated the effectiveness of weighted ensemble approaches combining time series and machine learning models for infectious disease prediction. This contradiction highlights the overall role of the forecasting granularity in determining how algorithms perform. In addition, our design of a weighted ensemble model, which is slightly less accurate than Random Forest, provides a valid framework for the integration of statistical and machine learning methods [18], consistent with our earlier findings that ensemble approaches can balance accuracy and stability [16]. This mixed approach gives a versatile basis, which can be further expanded through the integration of more data in the future, with the possibility of increased predictive ability as even more variables are available [47].

The methodological novelties of the given research are a breakthrough in the history of dengue prediction in Bangladesh. Unlike previous models by Karim et al. [48] and Dey et al. [49], which were limited by temporal aggregation and imprecise feature engineering, our framework uses high-resolution daily data, extensive feature engineering to include lagged variables and rolling statistics, and an advanced ensemble model. This approach builds upon our previous work applying machine learning to infectious disease forecasting [16, 17], extending these methodologies to dengue prediction in Dhaka. This holistic approach allowed our Random Forest model to predict with unprecedented accuracy (R^2^ = 0.95, RMSE = 34.64) - far beyond the 70–85% range of accuracy of earlier monthly models in Bangladesh and other endemic areas [50–52].

This work adds importantly to the evidence base for dengue transmission in the region and helps to refine public health practitioners’ understanding of the seasonal transmission pattern of dengue, helping inform their annual planning in urban cities with challenging conditions, like in Dhaka.

## Limitations of this study

There are a few weaknesses that should be taken into consideration when interpreting our findings. To begin with, this research involves the analysis of aggregated daily cases in the city of Dhaka, and, thus, is an ecological study, which is prone to ecological fallacy. Our predictions are not intended to be used in individual infection risk prediction, but to influence the city-wide public health planning. Second, the case information fails to differentiate between the residents and non-residents of Dhaka who come to the city to get care, since patient residence data are not provided in the Kaggle dataset. Therefore, our projections do not indicate the total caseload that will present to the health units in Dhaka, but instead the presentation of caseload based on local transmission only, and not exclusively on local transmission, but operationally in terms of health systems planning. Third, direct information on vector surveys (e.g., Breteau Index, larval density), virological aspects (distribution of serotypes), was not available at daily resolution. Fourth, we deterministically predict our ensemble, i.e., there are no formally prescribed prediction intervals, since the weighted combination of ensemble predictions does not naturally give uncertainty estimates. Fifth, the spatial heterogeneity, infrastructural (water and waste management) factors, and mobility are not reflected in our analysis at the city level. Lastly, this was a univariate time series study, and hence, no meteorological variables (temperature, humidity, rainfall) were included. Without these missing variables, our model is reflective of seasonal transmission patterns rather than truly predictive. Any significant changes in climate, population movement, or urban development in 2026 would cause important changes that our model cannot account for. True predictive capacity will require integration of these data types in future work.

## Future research

Based on this work, there are a number of directions that should be developed. To begin with, it is recommended that future research add the meteorological variables (temperature, humidity, rainfall, etc.) when possible, as these variables are known to impact the Aedes mosquito ecology and dynamics of the spread of dengue. Second, direct data on the surveillance of vectors particularly (Breteau Index) should be incorporated where available at a sufficient level of time, which may be done in conjunction with entomological surveillance programs in Dhaka. Third, case data disaggregated spatially would allow stratifying risks and intervention more precisely at a neighborhood level. Fourth, the origin of patients must be regularly taken to be able to differentiate between the local transmission and imported infections that seek care in Dhaka. Fifth, to be genuinely multifactorial, it would have to include elements of population migration, urbanization, changes in infrastructure (piped water coverage, waste management), and household water storage behavior. This would involve cooperation among health, municipal, and meteorological agencies. Lastly, there should be quantification of uncertainty with the bootstrap technique, quantile regression technique, or variance decomposition to equip the decision-makers with prediction ranges to help them respond to worst-case scenarios.

## Conclusion

This study demonstrates that machine learning and ensemble methods offer substantial advantages for dengue modeling in Dhaka. While Random Forest achieved superior point accuracy, the weighted ensemble, combining Random Forest with SARIMA, delivered an optimal balance between precision and forecast stability. The ensemble produced smoother, more epidemiologically plausible trajectories with substantially reduced day-to-day volatility, addressing a critical need in operational public health planning. Application of this framework to project the 2026 dengue season identified a distinct high transmission period beginning in early spring, which may help inform annual planning discussions and vector control timing. This work presents an important step forward toward data-driven preparedness in high-density urban settings. The ensemble approach, despite modest accuracy trade-offs, provides the stability and interpretability that may be useful for real-world applications, offering potential support for epidemic prevention and control efforts. With the future addition of missing data types (climatic, case origins, vector, and urban infrastructure data), a true outbreak forecasting framework can be developed. Such an expanded framework, when applied, could improve dengue outbreak forecasting in urban centers across dengue-endemic countries in the future.

## Electronic supplementary material

Below is the link to the electronic supplementary material.

Supplementary Material 1

Supplementary Material 2

## Data Availability

The dengue dataset used in this study is publicly available from Kaggle (https://www.kaggle.com/datasets/anikhasan8118/daily-dengue-cases-and-weather-dhaka-20212024). Processed data used for analysis are available from the corresponding author upon reasonable request. The necessary data and source codes are also available at https://github.com/Sakther12/Code_Dengue_Dhaka_1
