# Supplementary material for: Bridging ensemble model and public health practice: an approach for refining understanding of seasonal dengue transmission patterns in Bangladesh
Source: BMC Infect Dis. 2026 May 20;26:1331. doi: 10.1186/s12879-026-13573-3 (PMC13366745; doi:10.1186/s12879-026-13573-3)
Supplement: Supplementary file 2 — Supplementary Material 2 [file 12879_2026_13573_MOESM2_ESM.docx]

**SUPPLEMENTARY ANALYSIS: Data Preparation and Forecasting Procedure**

The original dataset comprised 1,826 daily observations from January 1, 2020 to December 30, 2024. Due to lag-30 feature construction requiring 30 preceding days, the first 30 days (January 1-30, 2020) were excluded, yielding 1,796 complete observations (January 31, 2020 to December 30, 2024). Zero values were retained as epidemiologically meaningful. A chronological 80/20 split produced 1,430 training days (January 31, 2020 to December 30, 2023) and 336 validation days (January 30, 2024 to December 30, 2024), with training median (48.0 cases) fixed for all subsequent steps to prevent leakage. For 2026 forecasting, we employed a recursive multi-step strategy where features were reconstructed at each step using only historical data and previously forecasted values. Lag features and rolling statistics were computed exclusively from available past data, and the threshold feature remained fixed to the training median. This approach ensures temporal validity and directly addresses data leakage concerns.

**SUPPLEMENTARY ANALYSIS: Random Forest vs Ensemble Comparison**

While the ensemble showed higher RMSE (44.32) compared to Random Forest (34.64) on the validation set, it offers important operational advantages. The ensemble reduces average daily volatility by 14.3% (from 20.02 to 17.16 cases) and, critically, eliminates all extreme day-to-day jumps (from 1 day to 0 days above the 95th percentile threshold). This smoothing effect is statistically significant (p < 0.001) and consistent across 59.2% of rolling 30-day windows.

For public health practice, this trade-off is justified: a 27.9% increase in point prediction error yields a 14.3% reduction in forecast volatility and complete elimination of extreme fluctuations. Stable predictions are essential for operational planning, as jagged forecasts could trigger unnecessary resource mobilization or erode stakeholder confidence. The ensemble thus represents a pragmatic choice for real-world application, balancing accuracy with the stability required for decision-making.


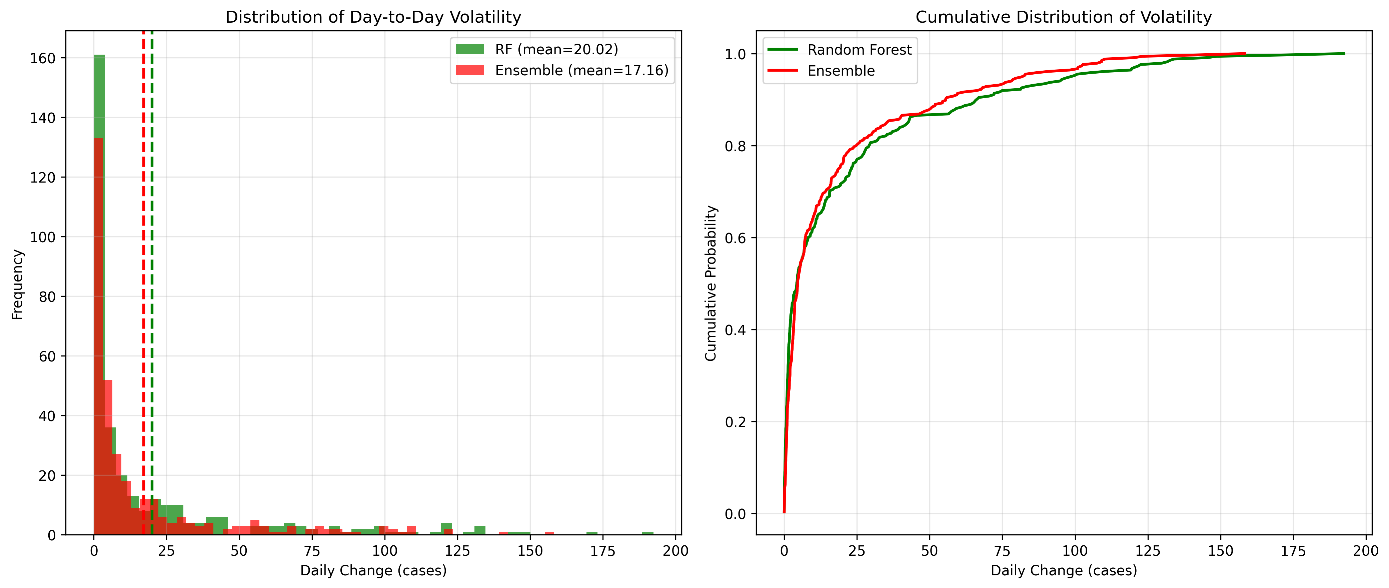


Fig S1: Random Forest vs Ensemble Comparison.

**Supplementary Material: Threshold Sensitivity Analysis for 2026 Dengue Forecast**

This supplementary material provides a detailed sensitivity analysis for the definition of "high transmission period" in the 2026 dengue forecast. We evaluated alternative percentile thresholds to assess the robustness of our primary choice (75th percentile). Using the ensemble forecast for 2026 (n = 365 days), we calculated case thresholds corresponding to the 70th, 75th, 80th, 85th, and 90th percentiles. For each threshold, we identified:

- The threshold value (daily cases)

- The start date of the high transmission period (first day exceeding the threshold)

- The duration (consecutive days above threshold)

- The excess of the peak over the threshold

Table S1. Sensitivity analysis of percentile thresholds for high transmission period identification.

| Percentile | Threshold (cases) | Duration (days) | Start Date | End Date | Peak Excess (cases) |
| --- | --- | --- | --- | --- | --- |
| 70th | 35 | 71 | 22-Feb | 3-May | 3 |
| 75th | 35 | 71 | 22-Feb | 3-May | 3 |
| 80th | 35 | 71 | 22-Feb | 3-May | 3 |
| 85th | 36 | 40 | 8-Mar | 16-Apr | 2 |
| 90th | 37 | 6 | 15-Mar | 20-Mar | 1 |

**Key Findings:**

- 70th-80th percentiles: All produced identical thresholds (35 cases) and identified the same 71-day high transmission period starting 22 February 2026.

- 85th percentile: Identified a 40-day period starting 8 March 2026 with a slightly higher threshold (36 cases).

- 90th percentile: Identified a 6-day period starting 15 March 2026 with the highest threshold (37 cases).

The stability of results across the 70th-80th percentile range demonstrates that our identification of February-March as the peak transmission window is robust to threshold selection. The 75th percentile was retained as the primary definition as it represents the upper quartile, a conventional cut-off for defining above-normal activity in epidemiological surveillance.

The modest peak excess (3 cases above the 75th percentile threshold) indicates that 2026 is forecast to be a mild dengue year, with sustained but not extreme transmission intensity. This is consistent with the relatively low predicted peak of 38 cases compared to historical maxima.

*"****Important note:*** *These percentile thresholds are statistical definitions based on historical data and do not correspond to official outbreak declarations used by public health surveillance systems. They are intended solely as research tools for identifying periods of elevated transmission risk within this study."*

**Interpretation for Public Health Planning**

The following interpretations are based on statistical thresholds and are intended to inform planning discussions; they do not constitute official outbreak declarations.

The sensitivity analysis reveals three important insights for operational planning:

1. **Robust peak window:** Regardless of threshold choice (70th-90th percentiles), February-March is consistently identified as the period of highest transmission.
2. **Mild year forecast:** The small excess of peak over threshold (+1 to +3 cases) suggests 2026 will be a mild dengue year, with no dramatic outbreak spikes.
3. **Threshold stability:** The identical results for 70th-80th percentiles indicate that public health officials can have confidence in the identified 71-day high transmission period, as it is not sensitive to the exact threshold chosen.

**SUPPLEMENTARY ANALYSIS: Engineered Predictors Table**

Table S2. Engineered Features for Machine Learning Models.

| **Feature Group** | **Feature Name** | **Description** | **Calculation** |
| --- | --- | --- | --- |
| **Lags** | Lag1 | Cases from previous day | Dengue_Cases[t-1] |
|  | Lag7 | Cases from 7 days ago | Dengue_Cases[t-7] |
|  | Lag30 | Cases from 30 days ago | Dengue_Cases[t-30] |
| **Rolling statistics** | Rolling_Mean_7 | 7-day moving average | mean of Dengue_Cases[t-7 : t-1] |
|  | Rolling_Std_7 | 7-day moving standard deviation | std of Dengue_Cases[t-7 : t-1] |
| **Calendar variables** | Month | Month of the year (1-12) | Extracted from date index |
|  | Month_1 to Month_12 | One-hot encoded month dummies | 1 if month matches, else 0 |
| **Threshold feature** | Cases_Threshold | Binary indicator for above-median cases | 1 if Dengue_Cases > training_median, els |

**Important note:** The training_median used for Cases_Threshold was calculated exclusively from the training period (January 31, 2020 - December 30, 2023) and fixed at 48.0 cases. This value was never updated during validation or forecasting to prevent data leakage.

**SUPPLEMENTARY ANALYSIS: TimeSeriesSplit Configuration**

Hyperparameter tuning for all machine learning models was conducted using expanding-window time series cross-validation implemented via TimeSeriesSplit(n_splits=3). The resulting splits are detailed below:

Table S3. Configuration of TimeSeriesSplit for hyperparameter tuning of machine learning models.

| **Split** | **Training indices** | **Training size** | **Validation indices** | **Validation size** | **Training end date** | **Validation start date** |
| --- | --- | --- | --- | --- | --- | --- |
| Split 1 | 0 – 358 | 359 | 359 – 715 | 357 | 2021-01-23 | 2021-01-24 |
| Split 2 | 0 – 715 | 716 | 716 – 1072 | 357 | 2022-01-15 | 2022-01-16 |
| Split 3 | 0 – 1072 | 1073 | 1073 – 1429 | 357 | 2023-01-07 | 2023-01-08 |

**Key features of the cross-validation scheme:**

- **Expanding window design**: Training set size increases progressively across folds (359 → 716 → 1073 observations), mimicking real-world forecasting where models learn from all available historical data up to the prediction point.
- **Strict temporal order**: In every fold, validation data occurs entirely after the training data, ensuring no future information leakage into the hyperparameter selection process.
- **Fixed validation size**: Each validation fold consists of exactly 357 consecutive observations (~1 year of daily data), providing consistent evaluation periods.
- **Chronological integrity**: The scheme fully respects the time ordering of the dengue incidence series, which is essential given the strong temporal autocorrelation and seasonality present in the data.
- **Index consistency**: All indices are zero-based and span the full pre-validation period (0 to 1429), corresponding to the training + tuning dataset before the final 2024 hold-out period.

This time-series-aware cross-validation strategy ensures that hyperparameter choices reflect realistic predictive performance on unseen future data, avoiding optimistic bias that would arise from random or non-temporal splitting methods. GridSearchCV (or an equivalent) was configured with cv=TimeSeriesSplit (n_splits=3) to enforce this structure throughout the tuning procedure.
